# Correlation between redefined optical coherence tomography parameters and best-corrected visual acuity in non-resolving central serous chorioretinopathy treated with half-dose photodynamic therapy

**DOI:** 10.1371/journal.pone.0202549

**Published:** 2018-08-24

**Authors:** Thomas J. van Rijssen, Danial Mohabati, Greet Dijkman, Thomas Theelen, Eiko K. de Jong, Elon H. C. van Dijk, Camiel J. F. Boon

**Affiliations:** 1 Department of Ophthalmology, Leiden University Medical Center, Leiden, The Netherlands; 2 Department of Ophthalmology, Donders Institute of Brain, Cognition and Behaviour, Radboud University Medical Center, Nijmegen, The Netherlands; 3 Department of Ophthalmology, Academic Medical Center, University of Amsterdam, Amsterdam, The Netherlands; Roskamp Institute, UNITED STATES

## Abstract

**Purpose:**

To assess parameters on optical coherence tomography (OCT), and their correlation with best-corrected visual acuity (BCVA) in patients with non-resolving central serous chorioretinopathy (CSC).

**Methods:**

For 25 non-resolving CSC patients treated with photodynamic therapy (PDT), the thickness of retinal layers was assessed on the foveal spectral-domain (SD) OCT scan. Evaluated OCT parameters included the central retinal thickness (CRT), defined as the internal limiting membrane (ILM) to ellipsoid zone (EZ) distance, and the second band thickness (SBT), defined as the EZ to hyperreflective subretinal accumulation distance. Integrity of the external limiting membrane (ELM) and the EZ bands was also determined. These parameters, along with BCVA and CRT measured automatically by SD-OCT device software were obtained before PDT, after PDT, and at final visit. After Bonferroni correction, a p-value <0.007 was considered statistically significant.

**Results:**

Twenty-five patients could be included at last visit before PDT and first visit after PDT. At final visit, 24 patients could be included, since 1 patients was lost to follow-up. Mean CRT was 112 μm at last visit before PDT, 118 μm at first visit after PDT (p = 0.030), and 127 μm at final visit (p<0.001compared to baseline). Mean SBT was 74 μm, 26 μm (p<0.001 compared to baseline), and 21 μm (p<0.001 compared to baseline), respectively. Mean BCVA in Early Treatment of Diabetic Retinopathy Study letters was 79 at baseline, 85 at first visit after PDT (p = 0.005 compared to baseline), and 87 at final visit (p = 0.001 compared to baseline). BCVA had an estimated correlation of β = 0.103 (p = 0.114) with CRT, β = -0.051 (p = 0.014) with SBT, β = 0.615 (p = 0.600) with the integrity of the ELM, and β = 4.917 with the integrity of the EZ (p = 0.001).

**Conclusions:**

In non-resolving CSC patients treated with half-dose PDT, the CRT increased at final visit in comparison to the last visit before PDT. The continuity of the EZ on SD-OCT was positively correlated with BCVA. We propose that the distance between ILM and EZ should be used as a reliable CRT measurement in non-resolving CSC patients treated with half-dose PDT.

## Introduction

Central serous chorioretinopathy (CSC) is characterized by a serous retinal detachment due to hyperpermeability of the choroidal vessels, subretinal leakage of serous fluid, and failure of the retinal pigment epithelium (RPE) in resorbing the excessive fluid accumulation.[1, 2] This process can lead to complaints such as vision loss, metamorphopsia, and changes in color and contrast vision. Several factors, such as male sex, use of steroid-containing medication, type A personality, and pregnancy are known to be associated with CSC.[3] Two forms of CSC have been described in literature, of which the overlap is currently unclear.[4] Acute CSC, which is characterized by a serous retinal detachment with limited focal or multifocal RPE alterations, usually recovers within a few months without the need of treatment.[4] Non-resolving CSC patients on the other hand, present with more extensive abnormalities in the macular area, without spontaneous recovery.[4] Half-dose photodynamic therapy (PDT) is often a successful treatment in non-resolving CSC patients, accelerating the resolution of subretinal fluid (SRF), with a favourable safety profile.[5] The effects of PDT may be long-lasting, causing permanent changes and possible damage to the choroidal vasculature, as shown in a primate model.[6] However, while PDT and micropulse laser treatment appear the most promising treatments for non-resolving CSC, consensus on the optimal treatment strategy in the management of non-resolving CSC is yet to be achieved, which may be a facilitated using the anticipated results of prospective randomized trials such as the PLACE trial.[7, 8] To date, optical coherence tomography (OCT) can be used both for diagnostic purposes and to evaluate the amount of SRF and the retinal anatomy before and after treatment.

**Fig 1:**
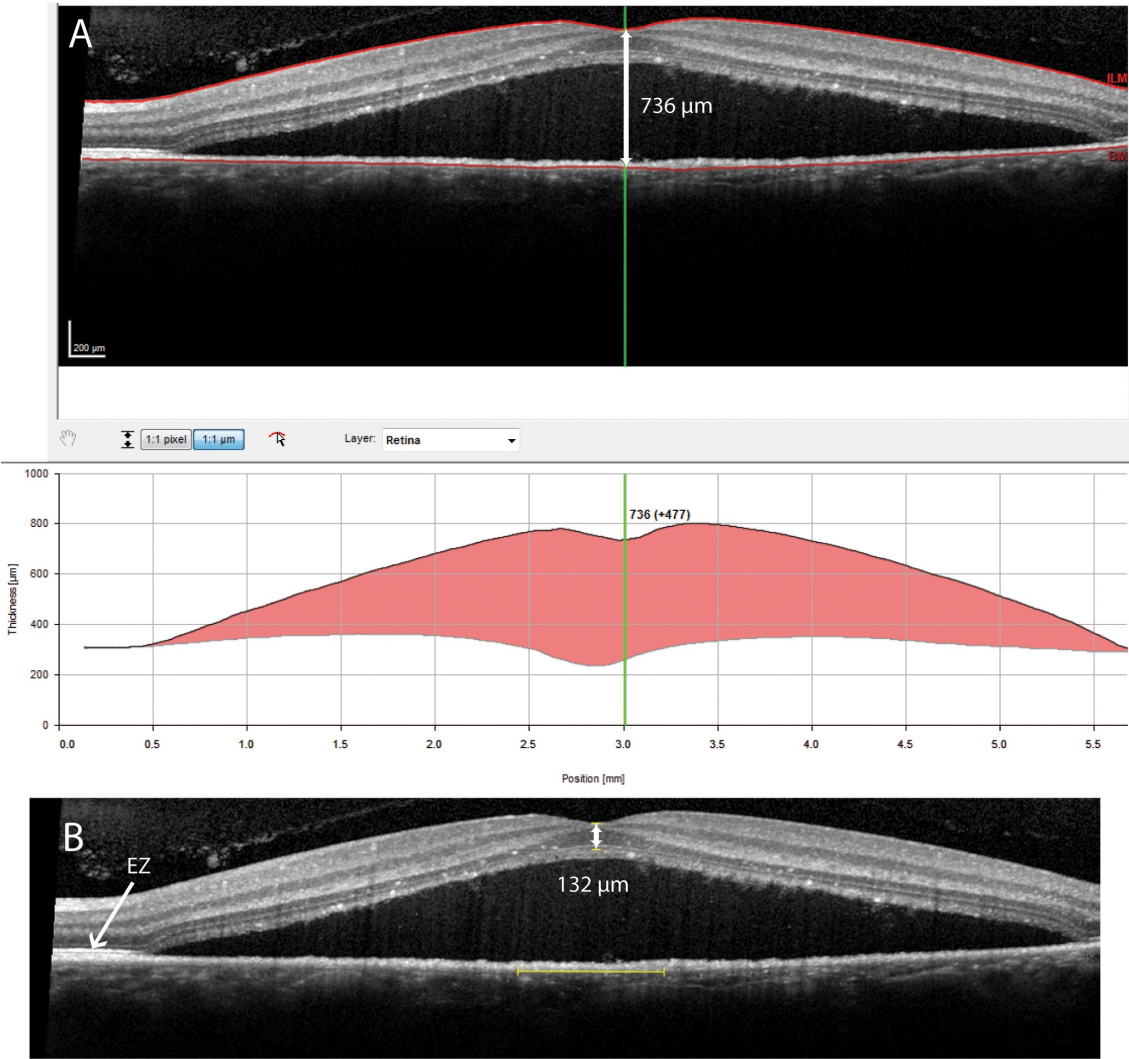
This figure shows an example of potential marked differences between automatic and manual central retinal thickness (CRT) measurements on spectral-domain optical coherence tomography (SD-OCT) in a patient with non-resolving central serous chorioretinopathy. A: Automatic CRT measurement by SD-OCT device software, in which the distance between the inner limiting membrane (ILM) and Bruch’s membrane was identified as the ‘CRT’ (736 μm), whilst erroneously including the subretinal fluid which obviously should not be part of a retinal thickness measurement. B: Manual CRT measurement in the same case, in which CRT was defined as the distance from the ILM to the inner border of the ellipsoid zone (EZ; 132 μm).

The thickness and integrity of the various retinal layers visualized on OCT might be of interest to predict visual outcome of a CSC episode. The central retinal thickness (CRT) is a commonly used anatomical parameter to evaluate treatment outcomes in the spectrum of common retinal diseases, such as age-related macular degeneration, diabetic macular edema, retinal venous occlusion, as well as CSC. The CRT in non-resolving CSC before and after PDT treatment and its relationship with best-corrected visual acuity (BCVA) has been evaluated in several retrospective studies.[9–13] However, there is currently no consensus how to measure CRT in CSC patients. For example, the distance between the inner and outer retinal surface has been used.[12] In other studies, the distance between the inner limiting membrane (ILM) and either the RPE or Bruch’s membrane (BM) was measured either manually or automatically by the OCT software (Fig 1).[9–11, 13–17] When defining the ILM to BM distance as CRT, a post-PDT decrease in CRT has been described that even exceeds the actual thickness of the neuroretina.[9] These CRT measurements may result in false representations of treatment results, due to the fact that these measurements erroneously include the SRF in automated measurements of CRT. This stresses the need for a universal definition of CRT, subretinal layers, and structures in CSC, both for documentation and possible prediction of therapeutic outcome.

Spaide and Curcio have previously linked different hyperreflective bands visualized on OCT to the histological anatomy of retinal layers.[18] The distance between the ILM and the EZ measured on spectral-domain (SD) OCT seems to approach the histological thickness of the neurosensory retina most accurately in patients with macular diseases with SRF accumulation. Relatively little is known about the correlation between CRT and other OCT parameters, and outcome of treatment in non-resolving CSC. Also, variable measurements and definitions of OCT parameters such as CRT have been described in non-resolving CSC in the past.[9, 16, 19] The aim of this study was to define CRT, thickness of the second hyperreflective band (SBT), integrity of the external limiting membrane (ELM), integrity of the ellipsoid zone (EZ) in non-resolving CSC patients, and to assess their correlation with the outcome of PDT treatment in this patient group.

## Materials and methods

This study adhered to the tenets of the Declaration of Helsinki. Written informed consent was acquired from all patients involved in the study. This study has been approved by the Central Committee of Human Research of the Leiden University Medical Center. Approval number: P14.297

The medical records and multimodal imaging data of 26 patients (26 eyes, 1 eye per patient) who were diagnosed with non-resolving CSC, also described as chronic CSC,[20] and who were treated with half-dose PDT between March 2014 and November 2016 at the Department of Ophthalmology of Leiden University Medical Center (Leiden, the Nederlands), were analysed. The diagnosis of non-resolving CSC was based on the presence of SRF > 3 months on SD-OCT, characteristic ‘hot spots’ of leakage, diffuse leakage, and RPE alterations visualized on fluorescein angiography (FA), and diffuse hyperfluorescent areas on indocyanine green angiography (ICGA). In the current study, only patients with foveal SRF at baseline were included, and this SRF had to be completely resolved after a maximum of 2 half-dose PDT treatments. Patients with either intraretinal fluid or suspicion of other retinal pathology, or patients who were previously treated for earlier episodes of CSC, were excluded. Patients in whom the retinal layers were insufficiently distinguishable due to suboptimal imaging quality of the SD-OCT scans, were also excluded.

For the PDT procedure, all patients received an intravenous infusion of 3 mg/m^2^ (half-dose) verteporfin (Visudyne®; Novartis, Basel, Switzerland) during an infusion time of 10 minutes. At exactly 15 minutes after the start of infusion, an anaesthetic eye drop was given (oxybuprocaine 0.4% or equivalent) before a contact lens (macula contact glass) was positioned on the affected eye. Subsequently, laser therapy with a standard fluency of 50 J/cm^2^ and a wavelength of 689 nm was applied to the area to be treated for 83 seconds. The laser spot size was chosen according to the area of hyperfluorescence on late phase ICGA, plus a treatment margin of 1 mm. If necessary, several laser spots were applied to cover the whole affected area.

All patients underwent BCVA measurement in Early Treatment of Diabetic Retinopathy Study (ETDRS) letters (Precision Vision, La Salle, Illinois, USA) at a distance of 4 meters, slit lamp examination, and multimodal imaging at the visit prior to PDT, and the visit(s) after PDT. In all patients, multimodal imaging was performed at baseline, including SD-OCT, fundus autofluorescence (FAF) imaging, FA, and ICGA (all acquired with Spectralis HRA+OCT, Heidelberg Engineering, Heidelberg, Germany). At final visit, the following examinations were performed: BCVA in ETDRS, slit lamp examination, SD-OCT, and FAF. FA and ICGA were only repeated in selected cases with persistent SRF accumulation at follow-up. The distances between different retinal and thickness measurements on the SD-OCT images were performed manually, in the 1:1 μm mode, using maximal (800%) magnification, and with use of the caliper tool in Heidelberg Eye Explorer, version 1.9.10.0. The Spectralis HRA+OCT has an axial resolution of less than 7 μm and uses a superluminescent diode with a center wavelength of 870 nm.[18, 21]

**Fig 2:**
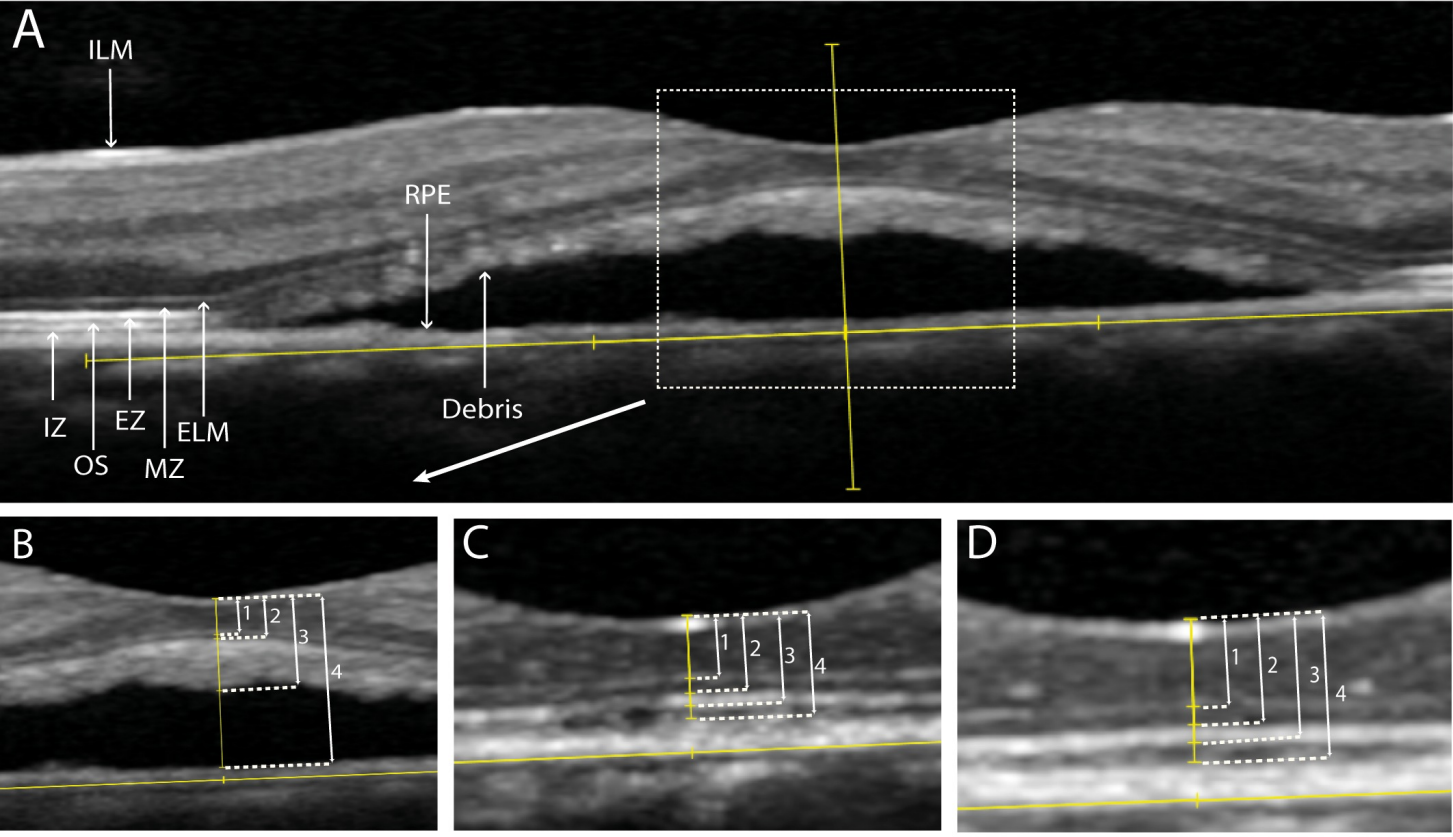
Retinal thickness measurements on spectral-domain optical coherence tomography (SD-OCT) in a patient with non-resolving central serous chorioretinopathy, who was treated with half-dose photodynamic therapy (PDT). Overview of the SD-OCT at the visit prior to PDT (A). Measurements at last visit before PDT (B). Measurements at the first visit after PDT (C). Measurements at final visit (D).

On SD-OCT, the distance from ILM to outer part of ELM, ILM to inner part of EZ, ILM to outer part of hyperreflective accumulation, and ILM to RPE were measured in the foveal pit at last visit before PDT, first visit after PDT, and final visit (Fig 2). In addition to the manual CRT measurement, CRT was also obtained through automatic measurement by the SD-OCT machine itself. The exact location of the foveal pit was recognized by both the maximal foveal dip and the hyperreflective aspect of the ILM, and the location of the outer nuclear layer curvature. First, a line was drawn based on the outer part of BM from the most nasal to the most temporal side of the SD-OCT scan with the foveal pit in the center of the scan. Second, a protractor was used to draw a line perpendicular on the first line, through the foveal pit (second line). The retinal measurements were done at the foveal pit, on the second line (Fig 2). After resolution of SRF, the foveal pit may have shifted. Therefore, the foveal pit was defined again, both at first visit after PDT and at final visit in order to perform the subsequent measurements.

Distance between the inner part of the internal limiting membrane (ILM) and the outer part of the external limiting membrane (ELM) (1). Distance between the inner part of the ILM and the inner part of the ellipsoid zone (EZ) (2). Distance between the inner part of the ILM and the outer part of the hyperreflective accumulation within the subretinal fluid (3). Distance between the inner part of the ILM and inner part of the retinal pigment epithelium (RPE) (4). Interdigitation zone (IZ), outer segments (OS) of the photoreceptors.

**Fig 3:**
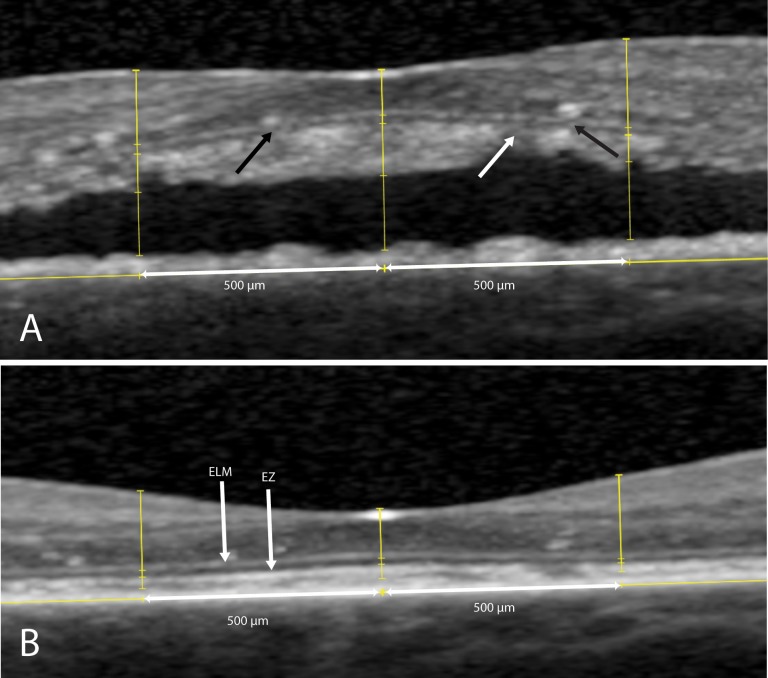
Integrity of the external limiting membrane (ELM) and ellipsoid zone (EZ) on spectral-domain optical coherence tomography in a patient with non-resolving central serous chorioretinopathy, who was treated with half-dose photodynamic therapy (PDT). At the visit prior to PDT, discontinuity is seen at the ELM (A, black arrows) and the EZ (A, white arrow) within 500 μm of the foveal pit (A). At the first visit after PDT, the ELM and EZ are both continuous within 500 μm of the foveal pit (B).

Evaluated OCT parameters in this study included: CRT changes over time, the change in foveal SBT over time, the correlation between BCVA and CRT, and the correlation between BCVA and SBT. Additionally, the correlation between BCVA and ELM integrity on SD-OCT, and the correlation between BCVA and the EZ integrity on SD-OCT were assessed. In our study and for manual measurements, we defined CRT as the distance from the inner border of the ILM to the inner border of the EZ on SD-OCT, as this may approach the true thickness of the neuroretina the most in non-resolving CSC cases which often accumulate subretinal debris. We are aware that this measurement lacks the EZ and outer segment thickness, which is also part of the retina. However, these layers are indistinguishable from the hyperreflective debris that is often seen within the SRF on SD-OCT in an active stage of non-resolving CSC (Fig 2A). The SBT was defined as the distance between the inner border of the EZ and the outer part of the debris when SRF was present, or the distance between the inner border of the EZ and the outer part of the EZ when the subretinal debris on SD-OCT had resolved after PDT. The value for SBT was not measured but calculated by subtracting the distance between ILM and inner border of the EZ, from the distance from ILM to the outer border of the hyperreflective subretinal accumulation of debris (Fig 2). Discontinuity of either the ELM or the EZ was defined as: 1. decreased signal intensity, or 2. absence of signal as compared to these ELM and EZ bands at a distance of more than 500 μm of the foveal pit, or 3. disruption of the ELM or EZ line within 500 μm from the fovea on SD-OCT (Fig 3).

Statistical analyses were performed using SPSS Statistics (IBM Corp. version 23.0. Armonk, New York, United States of America). Paired t-tests and McNemar tests were used to compare the parameters between last visit before PDT, first visit after PDT, and final visit. Generalised estimating equations (GEE) analyses were used to evaluate the correlation between BCVA and foveal measurements on SD-OCT, and integrity of foveal ELM and EZ. A Bonferroni correction was performed to adjust for multiple testing. With 7 measured parameters, a p-value of α = 0.05/7 (0.007) was considered statistically significant.

## Results

**Fig 4:**
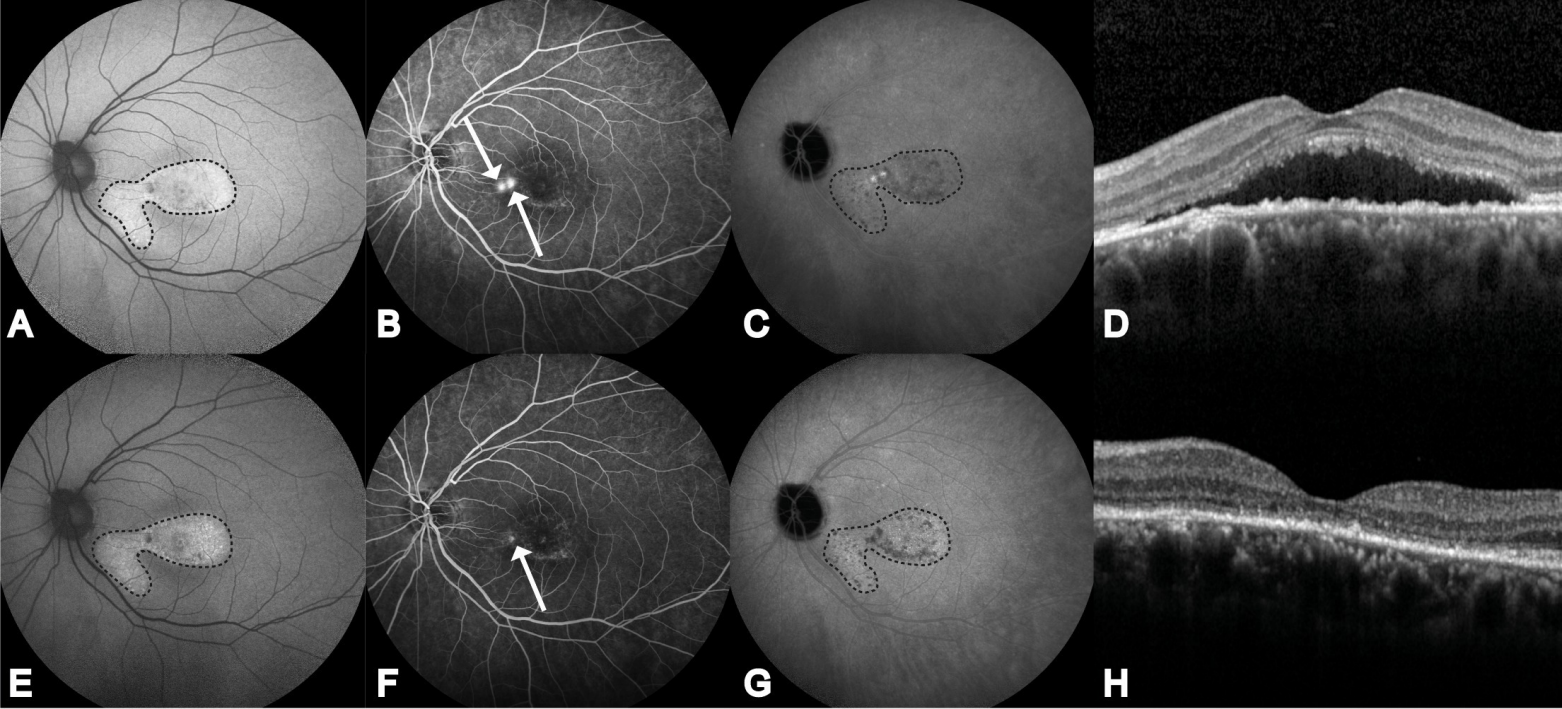
Multimodal imaging of the left eye of a 49-year-old male patient with non-resolving central serous chorioretinopathy. Fundus autofluorescence (FAF; A), fluorescein angiography (FA; B), indocyanine green angiography (ICGA; C), and optical coherence tomography (OCT; D) at last visit before half-dose photodynamic therapy (PDT). FAF (E), FA (F), ICGA (G), and OCT (H) at first visit at 10 weeks after PDT. Both hyperautofluorescent and hypoautofluorescent abnormalities are visible mainly in the fovea and nasal to the fovea on FAF (within the dashed borders; A). Two ‘hot spots’ of leakage on FA, nasal to the fovea (white arrows) and hyperfluorescent changes in the area below the fovea (B). Hyper- and hyporeflective abnormalities on ICGA (within the dashed borders), foveal and mainly in the area nasal to the fovea (C). Presence of foveal subretinal fluid (SRF) on OCT, with the presence of debris within the SRF accumulation (D). Abnormalities on FAF (within the dashed borders) did not change at the visit 10 weeks after PDT, compared to before PDT (E). One ‘hot spot’ of leakage is still present after PDT (arrow), with no obvious change in the hyperfluorescent areas that were already present (F). Hyper- and hyporeflective abnormalities showed subtle changes compared to before PDT (within the dashed borders) (G). A complete resolution of both SRF and hyperreflective subretinal debris was seen, with an improved integrity of the several neuroretinal layers (H).

Twenty-five out of 26 patients (21 males, 4 females) were included, and for these patients data prior to PDT, and for at least 1 visit after PDT were collected. No final visit was available for 1 out of the 25 included patients, because this patient was lost to follow-up. Additionally, 1 patient was excluded because of suboptimal quality of the SD-OCT images. There were 3 patients who received 2 PDT treatments, with an interval of 71 days, 48 days, and 69 days, respectively, between the PDT treatments. The mean age of the non-resolving CSC patients at the day of PDT treatment was 46.9 years (range, 33.4–62.8). Two patients had a retinal pigment epithelial detachment (PED) at baseline, which diminished during follow-up. The mean follow-up duration from PDT treatment until first visit after PDT, and until final visit was 1.7 months (range, 1.4–1.8) and 7.6 months (range, 6.8–9.4), respectively. The mean and range of the BCVA before and after PDT is summarized in Table 1. There was a mean improvement of 6 ETDRS letters (Snellen equivalent: 0.24) when comparing BCVA between baseline to first visit after PDT (p = 0.005), and a mean improvement of 8 ETDRS letters (Snellen equivalent 0.34) when comparing baseline to final visit (p = 0.001). An example of multimodal imaging characteristics of 1 patient at the visit before PDT and at first visit after PDT is shown in Fig 4.

**Table 1:** Best-corrected visual acuity (BCVA) and optical coherence tomography (OCT) parameters in non-resolving central serous chorioretinopathy (CSC) patients treated with half-dose photodynamic therapy (PDT).

	Last visit before PDT (n = 25)	First visit after PDT (n = 25)		Final visit (n = 24)		
	Mean (range)	Mean (range)	P-value	Mean (range)		P-value
BCVA in ETDRS letters	79 (58–90)	85 (63–96)	0.005	87 (57–93)		0.001
BCVA in Snellen equivalent	0.76 (0.29–1.26)	1.0 (0.36–1.66)	0.005	1.10 (0.28–1.45)		0.001
ILM to EZ distance in μm	112 (70–142)	118 (67–174)	0.030	127 (66–166)		<0.001
EZ to hyperreflective accumulation distance in μm (SBT)	74 (12–158)	26 (13–47)	<0.001	21 (13–36)		<0.001
CRT automatically calculated by OCT device software	396 (229–765)	230 (160–299)	<0.001	243 (164–300)		<0.001
	Number; percentage	Number; percentage		Number; percentage		
Continuous ELM layer	18 (72%)	21 (84%)	0.508	22 (92%)		0.125
Continuous EZ layer	0 (0%)	11 (44%)	0.001	17 (68%)		<0.001

BCVA, best-corrected visual acuity; ELM, external limiting membrane; ETDRS, Early treatment of Diabetic Retinopathy Study; EZ, ellipsoid zone; RPE, retinal pigment epithelium; ILM, internal limiting membrane; OCT, optical coherence tomography; SBT, second band thickness; SD, standard deviation. P-values compared to baseline visit.

The parameters that were assessed on OCT before and after PDT are summarized in Table 1. The CRT that was calculated automatically by the SD-OCT device software at the last visit before PDT was significantly higher than the manually measured CRT at last visit before PDT (p < 0.001). This was caused by the fact that the automated CRT measurement of the SD-OCT device erroneously included the SRF in all of the examined patients with SRF. The correlation of multiple parameters measured manually on SD-OCT with BCVA are summarized in Table 2. After performance of a GEE analysis, only excluding the final visit of the patient that was lost to follow-up, we observed an estimated correlation of β = 0.103 between an increase in CRT and an increase in BCVA at baseline and follow-up (p = 0.114). We found an estimated correlation of β = -0.051 between an increase in foveal SBT and an increase in BCVA (p = 0.014). No significant correlation was found between BCVA and the distance from ILM to ELM (estimated correlation β = 0.069; p = 0.341), the distance from ILM to hyperreflective subretinal accumulation (estimated correlation β = 0.042; p = 0.306), and the distance from ILM to RPE (estimated correlation β = -0.01; p = 0.014). The integrity of the ELM had an estimated correlation of β = 0.615 (p = 0.600) with BCVA, whereas this correlation was statistically significant between the EZ and BCVA (β = 4.917, p = 0.001).

**Table 2:** Correlations of best-corrected visual acuity (BCVA) and parameters visible on optical coherence tomography (OCT) in non-resolving central serous chorioretinopathy (CSC) patients treated with half-dose photodynamic therapy (PDT).

	Estimated correlation coefficient with BCVA in ETDRS letters (GEE analyses)	p-value*
ILM to EZ (CRT)	0.103	0.114
EZ to hyperreflective accumulation (SBT)	-0.051	0.014
ILM to RPE	-0.010	0.067
ILM to hyperreflective accumulation	0.042	0.306
ILM to ELM	0.069	0.341
Integrity of the ELM	0.615	0.600
Integrity of the EZ	4.917	0.001

BCVA, best-corrected visual acuity; CRT, central retinal thickness; ELM, external limiting membrane; ETDRS, Early Treatment of Diabetic Retinopathy Study; EZ, ellipsoid zone; GEE, generalized estimating equations; ILM, internal limiting membrane; SBT, second band thickness; RPE, retinal pigment epithelium;

* unadjusted p-values, after Bonferroni correction p-values < 0.007 were considered significant.

## Discussion

The CRT can be considered as an important parameter to evaluate the disease course in non-resolving CSC patients. However, there is currently no widely accepted definition of the CRT and there are multiple measurement techniques for CRT.[9, 16, 19] In this study, we introduced a definition of CRT in active non-resolving CSC patients, and evaluated multiple clinical findings on SD-OCT in patients treated with half-dose PDT. We defined CRT in active non-resolving CSC patients as the distance between inner part of the ILM and inner part of the EZ. In this study, we found a significant increased BCVA at first visit after PDT and final visit, increased CRT at final visit, and decreased SBT after treatment with PDT, compared to values before PDT. We did not find a significant correlation between the increase of CRT and improvement of BCVA. However, a positive correlation between the continuity of the EZ and improvement of BCVA was found.

We used generally accepted observations and definitions of retinal layers on SD-OCT that were described by Spaide and Curcio to correlate our observations on SD-OCT with retinal histology.[18, 22] In contrast to our results, other authors have described a decrease in CRT after resolution of SRF.[9–12, 14, 16, 17] Some studies have used the CRT values that were automatically measured by SD-OCT or the time-domain OCT software in patients who had SRF.[9–12, 14] This methodology appears to be inaccurate, since these measurements often include SRF, subfoveal PEDs, or subretinal debris, which should not be considered to be part of the neuroretina (Fig 1). As these incorrect automated CRT measurements are dependent on the resolution of SRF during follow-up, they do not give reliable information about the neuroretina itself. A complete resolution of SRF should be the primary goal of PDT treatment, which can for instance be evaluated during follow-up as height and/or volume of the SRF collection, but automatic CRT measurement is not useful for this purpose.[8, 11, 23] The potential problem of relying on automated CRT measurements in CSC is further illustrated by studies that have even described a reduction in CRT after SRF resolution that was larger than the average CRT in healthy individuals.[9, 16] Therefore, we propose to define CRT in active CSC and other macular diseases associated with serous subretinal fluid as the distance between the ILM and upper border of the EZ band as observed on SD-OCT (Figs 1 and 2).

The increase in CRT that we observed at final visit might be caused by restoration of the outer nuclear layer in the foveal pit after resolution of SRF, which has previously been suggested by Ojima et al.[24] In a healthy retina, photoreceptor outer segments are continuously renewed and the shed outer segments are phagocytized by the RPE that is tightly associated with the outer photoreceptors through apical microvilli that surround the photoreceptor outer segments. In active CSC, the photoreceptor outer segments are detached from the RPE microvilli, disturbing the normal anatomical junctions, which consequently impairs normal phagocytosis of photoreceptor outer segments. This may lead to elongation of the photoreceptor outer segments and accumulation of unphagocytosed photoreceptor outer segment debris that can contain autofluorescent lipofuscin precursors.[10, 25, 26] A thickened layer of shed unphagocytosed photoreceptor outer segment debris dangling below the neuroretina presumably is the main reason for the increased SBT we found prior to PDT compared to the SBT after PDT. With the resolution of SRF, reconstitution of the close anatomical and physiological relationship of the RPE microvilli with the outer parts of the photoreceptors will gradually occur. This will facilitate the phagocytosis of the photoreceptor outer segment debris by the RPE, with a subsequent gradual decrease in SBT. This is supported by the observations we made in our current study. After resolution of SRF, the visual outcome in CSC patients is relatively favourable compared to other retinal diseases.[4, 27] This may be due to the specific properties of the SRF in CSC, which consists of a relatively constant composition that may probably still allow some metabolic exchange and also provides a compartment in which photoreceptor outer segments can be shed. Also, the foveal cones receive metabolic support of Müller cells with which they have an alternative visual cycle for visual pigment regeneration that is independent of the main visual cycle via RPE that is interrupted in the case of CSC due to subfoveal SRF accumulation.[28, 29] In this alternative visual cycle, Müller cells process cone-derived all-trans retinol.[29] In the rod visual cycle, the RPE recycles the rod-derived all-trans retinol.[29] In this study, we found ELM discontinuity in a noteworthy number of non-resolving CSC patients. According to Spaide and Curcio, the ELM consists of Müller cell junctional complexes.[18] Discontinuity of the ELM may be a reflection of damaged Müller cells, leading to more impairment of the alternative visual cycle and worse BCVA, which is supported by other studies.[15, 30] However, in the present study restoration or persistent discontinuity of ELM integrity during follow-up was not significantly correlated with BCVA. A disruption of the EZ has been associated with an impairment of BCVA in CSC and other diseases.[15, 24, 30, 31] Ojima et al. have hypothesized that discontinuity of the inner segments/outer segments line (IS/OS, a term that was previously used for EZ) may be due to degeneration of the photoreceptor outer segments in the foveal photoreceptor layer, without permanent damage to the photoreceptor cells themselves.[24] This degeneration without permanent damage to the photoreceptor cells could lead to a recovery of the IS/OS (EZ) line after resolution of SRF.[24] Other studies have shown that the integrity of the IS/OS (EZ), or outer photoreceptor layer on time-domain OCT can be a good initial predictor of BCVA in CSC patients.[15, 24, 30, 32] In the current non-resolving CSC cohort, we did find a significant correlation between the integrity of the EZ and BCVA during follow-up, which is in line with the aforementioned studies.

There are limitations to this study. Despite this being a prospective cohort study, there is a minor difference in follow-up duration between each patient. This duration may have had an effect on the remodelling of the choroid. Within our definition of CRT in non-resolving CSC patients with active SRF, the EZ and photoreceptor outer segments, with an approximate distance of 55 μm in the healthy undetached fovea, will not be included.[18] In non-resolving CSC, which is virtually always associated with a certain degree of accumulation of subretinal hyperreflective debris, SD-OCT cannot distinguish the EZ and photoreceptor outer segments from the hyperreflective subretinal debris, which have comparable OCT reflectivity characteristics. Therefore, in order to accurately assess the CRT in non-resolving CSC patients treated with half-dose PDT, measurement of the distance between ILM to the inner part of the EZ may be inevitable. The assessment of SD-OCT parameters in this study was performed manually, and only BCVA was used as a clinical outcome measure. There were 3 patients in this study that received 2 PDT treatments. It is unknown to what extent a second PDT treatment may have had an effect on the evaluated OCT parameters. The BCVA in ETDRS letters represents the health status within an area of approximately 25 μm in diameter of the foveal pit. The CRT measurements in this study where performed at 1 point in the foveal pit of approximately 1 μm in diameter. The correlation of CRT with BCVA may improve with measurement of the area on OCT (circle with a diameter of 25μm, with the foveal pit in the center) that would represent the BCVA. In addition, the sensitivity of the SD-OCT device is less than 7μm.[18] This sensitivity may be too low to accurately observe changes in the retinal thickness layers. Also, the results of this study may only apply to non-resolving CSC patients without extensive atrophic RPE changes, also known as diffuse retinal pigment epitheliopathy, since a reduction of the SBT may lead to a poor BCVA in patients with such extensive changes.[23]

In conclusion, in non-resolving CSC patients, CRT is not significantly correlated with BCVA, while the SBT is negatively correlated with BCVA. CRT values that are obtained automatically by SD-OCT device software can be inaccurate and should therefore be used with caution, since these may include the SRF in CRT the measurement and can consequently lead to measurement errors in for instance studies on treatment outcome in non-resolving CSC. We propose that the distance between ILM and EZ, measured in the foveal scan on SD-OCT, should be used as a reliable CRT measurement in non-resolving CSC patients successfully treated with half-dose PDT.

## Supporting information

S1 Dataset Dataset that was used for the analyses. (SAV)
